# Sports injury and stressor-related disorder in competitive athletes: a systematic review and a new framework

**DOI:** 10.1093/burnst/tkac017

**Published:** 2022-06-11

**Authors:** Sophie Xin Yang, Siyu Cheng, Diana Linyi Su

**Affiliations:** Strategy and Human Resources of Business School Sichuan University, Wangjiang Campus, Sichuan University, Wuhou District, Chengdu, Sichuan 610065, China; Business Administration of Business School Sichuan University, Wangjiang Campus, Sichuan University, Wuhou District, Chengdu, Sichuan, China; Faculty of Education and Human Development of Early Childhood Education The Education University of Hong Kong, 10 Lo Ping Road, Tai Po, New Territories 999077, Hong Kong

**Keywords:** Stressor-related disorder, PTSD, Sports injury, Athletes

## Abstract

**Background:**

For professional athletes, sports injury has been considered one of the most influential factors determining their athletic careers' duration and quality. High-intensity training and competitiveness of the sports competition are perhaps critical causes of sports-related stress. This article reviews the relevant research on sports injuries and stressor-related disorders. Further, it explores the following three issues in depth: (1) Do physical injuries caused by competitive sports lead to acute or posttraumatic stress disorder for athletes? What are the abnormal stress responses? (2) What diagnoses are currently available for sports injury related traumatic stress disorder? (3) What kinds of psychological rehabilitation are available for trauma-related symptoms in sports injury? How efficient are they in alleviating these symptoms?

**Methods:**

The study searched electronic databases, including PubMed, MEDLINE, CINAHL, etc. And reference lists of included papers were also screened. Two researchers selected the literature strictly according to the inclusion criteria and sorted them out. Based on the proved conclusions, the study established a new framework to manage traumatic stress disorders after the injury occurred.

**Results:**

16 articles were included in the study. (Q1: N = 10; Q2: N = 3; Q3: N = 3 ) The findings of this review suggested that athletes who suffer from sports injuries are more likely to experience abnormal physiological or psychological stress responses, which may become a massive challenge for athletes to continue their sports careers at a competitive level. However, there is a minimal understanding of addressing sports injury-related traumatic stress disorder from a biological perspective. Thus, it is challenging to build a scientific basis for diagnosis, screening, and treatment. In addition, the current diagnostic tool for athletes stress disorder still heavily relies on subjective measurement, and the treatment plan is not different from that of the general population.

**Conclusions:**

It highlighted that sports-related stress disorder could be the greatest challenge to return to competition for injured athletes. The present study indicated the importance of systematically identifying the symptoms of sports-related stress disorder and improving the current diagnosis and treatment system.

Highlights

- Athletes who suffered from sports injury are more likely to experience abnormal physiological or psychological stress responses, and the severity may be related to the individual characteristics and injury events' characteristics.
- The treatments for stress disorders in injured athletes is no different from those in the general population, and the effectiveness of the current therapy remains to be further explored.
- The present study attempted to develop a new management framework for the traumatic stress disorders after injury, suggesting that personal files should be established to identify high-risk groups. After injury, abnormal symptoms can be detected early through a comprehensive screening management process. Then, according to types of sports, types of sports injuries and injury event characters, targeted interventions should be taken.

## Background

Injury is the biggest enemy for professional athletes. No matter how talented athletes are, they cannot avoid the occurrence of injuries. Consequently, injury can end their athletic career. Furthermore, physical as well as mental injuries, such as traumatic psychological disorders, may accompany them lifelong. Athletes who had to retire at their peak performance level because of injuries described the injury experience as a nightmare surrounding their daily lives [1]. Research on athletes who suffered from knee anterior cruciate ligament (ACL) injury also described overcoming the psychological barriers as being as complex as the physical function recovery during rehabilitation [2].

Traumatic stress disorder is seen as a severe psychological disorder, and research has indicated that athletes have a much higher possibility of suffering from stress disorders than the general population [3]. This is due to the frequency of experiencing physical injuries in sports [4]. The most common symptoms of sports injury-related stress disorder include attention distraction and uncontrolled body movements in specific scenarios [5], which become an obstacle preventing the athletes from returning to competitive sports. Furthermore, even when they are able to continue training and competition, the probability of re-injury may also increase [6].

Stress is a complex psychological process that includes the dynamics between the individual and environmental factors. For example, a soccer player who experienced medial collateral ligament (MCL) and ACL rupture constantly recalled the incident of injury while watching soccer games [7]. When individuals encounter a stimulus event that interferes with their internal balance or exceeds their ability to cope with it, they are more likely to experience a specific reaction process, including stress stimulus, perceptual evaluation of threat and stress response. Athletes with a history of injury tend to exhibit extreme cognitive judgment and a series of abnormal stress responses when facing a situation similar to the injury scenario (stressors) [8]. If this kind of reaction is severe and lasts for a long time, it may meet the diagnostic criteria of trauma- and stressor-related disorder.

According to the Diagnostic and Statistical Manual of Mental Disorders (DSM-5), the symptoms of trauma and stressor-related disorder, including acute stress disorder (ASD), post-traumatic stress disorder (PTSD) and adjustment stress disorder (AD), are intrusion, avoidance, dissociation and alterations in reactivity. ASD refers to the abnormal stress symptoms that people show within 2 days to 4 weeks after witnessing or experiencing traumatic events with death threats or severe injuries, such as natural disasters, traffic accidents and major diseases [9]. PTSD is a delayed and long-lasting stress disorder stimulated by similar events. The onset time is primarily within 1 month to 6 months after the event [10]. Empirical studies suggested that ASD has a specific predictive effect on PTSD—indicating that early detection of ASD symptoms may reduce the incidence of PTSD [11]. AD is a sort of mental illness that does not take the source of stress into account. Psycho-social stress and life changes are generally the leading causes of ASD [12], such as job changes or parental divorce. The present research targeted the scope of ASD and PTSD, since sports injury is similar to a catastrophic event for professional athletes [13].

To enhance the understanding of the relationship between sports injuries and stress disorders and establish more effective clinical guideline, this article will use the terminology of stress disorder to describe sports injury-related psychological disorders. The article is structured as follows. First, we examine the following specific issues. Do physical injuries caused by competitive sports lead to ASD or PTSD? What types of abnormal stress responses may appear? Second, we review and discuss the current diagnostic tools for sports injury-related traumatic stress disorder. Third, we focus on the type of rehabilitation that has proved efficient for trauma-related symptoms in sports injury. Finally, based on the results, we present a new traumatic stress disorder management framework for injured athletes.

## Methods

### Literature searches

This study searched electronic databases, including PubMed, MEDLINE, CINAHL, Sportdicus, Scopus, Psyinfo, CNKI and Wanfang; the reference lists of included papers were also searched.

The research question is divided into keywords: athlete, athletic injury, stress disorders, and diagnosis and psychological therapy. On this basis, the search terms were extended to sport(s) injury, sport(s) injuries, athletic injuries, traumatic, posttraumatic, posttraumatic stress disorder, PTSD, acute, acute stress disorder, ASD, stress disorder(s), measurement, instrument, psychological treatment, psychological remedy, and psychological therapeutics. Combinations of these search terms were used when conducting online searches.

### Selection criteria

We selected the publications according to the following criteria.

(1) Participants: competitive athletes, no age limits.
(2) Definition: sports were defined as competitive sports, including professional competitive sports, winning competitive sports. Sports injury refers to the physical injury caused by competitive sports.
(3) Method: the research related to question 1 (Q1) below requires that the phenomenon’s existence be proved quantitatively. The research related to questions 2 and 3 (Q2 and Q3) can be accepted by qualitative or quantitative methods.
(4) Contents: literature related to at least one research question. It is necessary to indicate sports injury, which is the only source of stress, and discuss different types of stress reaction or anxiety disorder shown by the athlete population. This study will attempt to avoid analysing mental health problems in general. Furthermore, psychological treatments (Q3) must be carried out in injured athletes.

### Screening and quality assessment

**Figure 1.**
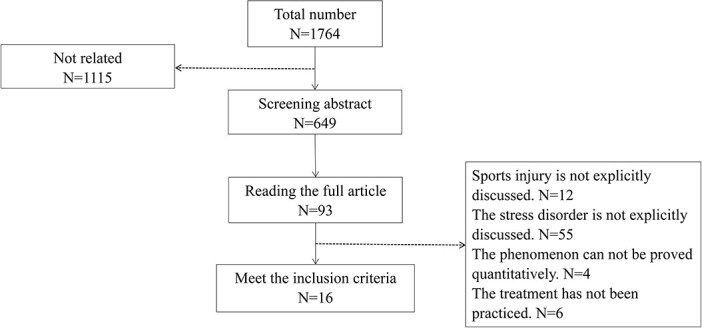
Screening flow chart

The primary literature search was completed in March 2021. Two researchers conducted literature screening and quality assessment according to the inclusion criteria (Figure 1). First, we browsed the titles and abstracts of articles for preliminary screening. Then, if the article met the group criteria, we further browsed the full text to judge the correlation. The literature was only incorporated when both researchers unanimously agreed to accept. If two researchers’ decisions are inconsistent, a third researcher will be asked to review and decide. In January 2022, we conducted a literature search again to supplement the relevant research in 2021, and the process was consistent. The criteria of literature quality evaluation followed mature protocols [14].

## Results

### Summary of included papers

Searches identified 1764 articles after removing duplicates. After preliminary screening of topics and abstracts, 93 articles were put forward for full-text screening. Of these, 77 were excluded for the following reasons: the sports injury was not explicitly discussed (*n* = 12), the stress disorder was not expressly discussed (*n* = 55), the phenomenon could not be proved quantitatively (*n* = 4) and treatment has not been carried out (*n* = 6).

Finally, 16 articles were included in the study. They were divided into three groups according to the research questions: the study to verify the relationship between sports injury and stress disorder (*n* = 10), research on the diagnosis of stress disorders in athletes (*n* = 3) and research related to treatment (*n* = 3).

Of the 16 papers included, 13 scored in the middle and upper level based on the quality evaluation scale. The scoring details are provided in the supplementary material, available online.

**Table 1:** Researches related to question 1

**Reference**	**Age** **(**years)	**Sample**	**Measurements**	**Whether it causes stress disorder**	**Symptoms and characteristics of stress disorder**	**Factors mentioned**
Shuer ML et al. 1997 [13]	Average= 19.5	280 Men 137 Women 143	IES	Athletes with acute and chronic sports injury showed symptoms of stress disorder.	(1) There was no significant difference in the score of intrusion symptoms between the chronically injured athletes and the fire victims. (2) The score of avoidance symptoms of long-term injured athletes was significantly higher than that of fire and earthquake victims. (3) There was no significant difference in avoidance behaviour score, between patients with bone injury caused by accident and athletes with acute and chronic injuries.	Sex
Newcomer RR et al. 2003 [18]	13–18	283 Men 143 Women 140	IES	The IES score of injured athletes increased by 35%–49% before and after the injury.	Athletes with a recent history of injury show more serious invasive thinking and avoidance behaviour.	Age
O’Neill DF 2008 [22]	13–19	459 Men 277 Women 182	Interview PANAS Injury contagion and performance testing	After witnessing the injury, teammates showed PTSD related symptoms.	(1) Fear of injury increased when teammates were injured. (2) Female athletes’ emotional swings were more pronounced after teammates were injured.	Sex
O’Connor JW 2010 [25]	18–30	35 Men 13 Women 22	TSI Brief COPE Inventory	Over 1/3 of the injured athletes had TSI scores over 65, the correlation between sports injury and posttraumatic distress exists.	Coping styles and injury severity were not related to the intensity of traumatic symptoms.	Coping style Injury severity
Edmed SL et al. 2015 [29]	/	122 Men 40 Women 82	Sport-mTBI vignette NSI PCL-C IPQ-R Perceived injury desirability	14.6% athletes without diagnostic, 20% athletes diagnosed with a concussion, and 19.5% athletes diagnosed with mTBI expected more significant PTSD symptom disturbance than the clinical cut-off in the control after reading the Sport-mTBI vignette.	There was no significant difference in expected PTSD symptoms when diagnosed with mTBI or concussion, or given no diagnosis.	Diagnosis
Xu S et al. 2018 [24]	/	268	The PTSD self-rating scale The international personality item pool- five-factor model measure SCSQ Colorado injury reporting system	There is a significant correlation between the degree of sports injury and stress disorder severity.	(1) The negative coping style plays a complete mediating effect between the degree of sports injury and posttraumatic stress disorder. (2) The mediating effect of negative coping style between the degree of injury and stress response is moderated by neuroticism personality.	Coping style Personality The severity of injury
Brassil HE et al. 2018 [26]	18–24	124	PC-TSS Self developed scale based on DSM-V	The score of posttraumatic stress disorder in the concussion group was significantly higher than in the healthy group. The score of stress disorder symptoms were increased considerably after injury.	For athletes with a history of sports-related concussion injuries, the most severe problem is a sleep disorder, followed by avoidance symptoms.	Type of sports injuries
Padaki AS et al. 2018 [19]	14.5±2.7	24 Men 12 Women 12	AIMS IES-R	After a sports injury, more than 80% of athletes showed symptoms related to stress disorder.	(1)After ACL rupture, more than 87.5% of the athletes had avoidance symptoms, 83.3% admitted to avoidance symptoms, and 75% had hyperarousal symptoms. (2)ACL ruptured athletes aged 15–21 have more severe symptoms of posttraumatic stress disorder than young athletes under 14 years of age. (3) The emotional trauma of female patients is more significant than male patients. (4) Patients with high athletic identities experienced greater emotional trauma than those with lower identities. (Not statistically significant)	Age Sex Identity
Bateman A et al. 2019 [23]	18–24	46 Men 30 Women 16	IES-R GSES	Sports injury has a specific predictive effect on posttraumatic stress disorder, especially for hyperarousal symptoms.	The results show that self-efficacy does not affect the development of PTSD.	Self- efficacy
Appaneal RN et al. 2007 [16]	19–25	12 Men 12	HR EDA(SCL SCR) BAM SUDS IES POMS LESCA	When watching a video of an injury, the physical and psychological stress responses of the athletes with a history of injury were significantly higher than the healthy group.	Injured athletes’ skin conductivity response and psychological stress pain were significantly higher than the control group.	/

*IES* Impact of Event Scale, *IES-R* Impact of Event Scale – Revised, *PANAS* Positive and Negative Affent Schedule, *PTSD* post-traumatic stress disorders, *TSI* traumatic stress inventory, *PCL-C* PTSD Checklist-Civilian Version, *mTBI* mild traumatic brain injury, *NSI* neurobehavioural symptom inventory, *IPQ-R* Revised Illness Perception Questionnaire, *SCSQ* Simplified Coping Style Questionnaire, *PC-TSS* post-concussion total symptom scores, *DSM-V* diagnostic and statistical manual of mental disorders, *AIMS* Athletic Identity Measurement Scale, *GSES* General Self-Efficacy Scale, *HR* heart rate, *EDA* Electrodermal Activity, *SCL* skin conductance level, *SCR* skin conductance response, *BAM* brief assessment of mood, *SUDS* Subjective Units of Distress Scale, *POMS* profile of mood states, *LESCA* Life-event Scale for Collegiate Athletes

### Q1. Does physical injury caused by competitive sports lead to ASD or PTSD? What types of abnormal stress responses may appear?

A multi-dimensional systematic analysis that included 10 quantitative studies was conducted to establish the link between the the stress response and diffculties after a sports injury. The total number of participants in the study was 1653 aged between 13 and 30 years, including youth athletes and high-level athletes, involving multiple types of sports, such as skiing, swimming, volleyball, soccer and basketball.

#### PTSD symptoms

As shown in Table 1, nine quantitative studies indicated that psychological symptoms related to traumatic stress disorders might include avoidance, hyper-arousal, sleep disorder and fear after injury. Psychological imbalance is often accompanied by physiological problems [15]. One study monitored athletes’ stress response through physiological indexes. The comparison between the athletes with or without injury history was made based on their heart rate (HR), skin conductivity level (SCL) and skin conductivity response (SCR). Results indicated that, when watching the related video of the injury, the SCR of athletes with a history of injury was significantly higher than that of healthy athletes [16]. Thus, the literature supported the conclusion that athletes would experience abnormal physiological or psychological stress responses after injury. While the presence of symptoms did not diagnose stress disorder in athletes, it suggested a possibility that needs to be paid attention to and managed. Research has shown that the effects of sports injury-related stress disorders were equivalent to those of earthquakes and fires. For instance, chronic sports injury may cause athletes to experience more severe avoidance symptoms than fire and earthquake victims. Athletes with intrusion symptoms tend to have similar experiences to fire victims [13].

### Factors determining PTSD: individual characteristics

Factors affecting the severity and symptoms of stress disorder can be divided into individual and injury events characteristics. Previous studies mainly discussed individual characteristics from demographic and psychological perspectives. However, research in the general population suggested that the morbidity and severity of PTSD are related to age, indicating that older adults may develop more vital emotional regulation skills than young people. Specifically, they could cope with negative emotions better [17].

Meanwhile, the researchers believed that child or adolescent athletes (13–18 years old) were more sensitive to trauma events and could experience more pain than adults. For instance, injured adolescent athletes expressed more intrusive thoughts and avoidance behaviours than those without a recent injury history. It is also reported that the impact of event (IES) score of these athletes increased by 35–49% before and after the injury [18]. However, a review was put forward that contradicted this conclusion to some extent. Findings showed that athletes aged between 15 and 21 years had more severe symptoms of PTSD than those under the age of 14 years, as older athletes may be more sensitive to the costs of surgery and the implications of missing a competitive season [19]. Regarding sex, women always tended to experience more severe symptoms than men in some major PTSD domains [20]. Empirical studies reported that stress disorder incidence in women was 2–3 times higher than in men [21]. Further, female athletes tended to have higher emotional fluctuations and trauma than male athletes in the same situation [22]. Oxytocin may be responsible for this difference [21]. More recently, researchers attempted to identify the psychological factors that influence the relationship between sports injuries and stress disorder, e.g. self-efficacy [23] and coping style, in which negative coping style showed a full mediation effect between the degree of sports injury and post-injury stress disorder [24]. Another study revealed contradictory findings, showing that coping styles were not related to the intensity of traumatic symptoms [25].

Additionally, athletic identity plays an important role. The stronger of the sense of athlete identity, the severer of the impact of injury, and the emotional effects may extend to PTSD [19]. In addition to the factors mentioned above, many other psychological characteristics predict traumatic stress disorders. The mechanism of the effect of sports injury on traumatic stress disorder still needs to be confirmed.

#### Factors determining PTSD: injury events’ characteristics

Other studies focused on the characteristics of injury events. For instance, different physical injuries may contribute to various stress disorder symptoms. Researchers conducted a study on concussion injury caused by sports. They found a significant difference in the level of stress disorder between the concussion group and the healthy group. Moreover, the trauma-related scale score of athletes after the injury was significantly higher than that before the injury [26]. ACL rupture of the knee is one of the most common sports injuries for athletes [27], especially in female athletes. An empirical study of high-level juvenile athletes showed that >87.5% had avoidance symptoms after ACL rupture, 83.3% admitted to intrusion symptoms and 75% had hyperarousal symptoms [19]. The two studies discussed above indicate the differences in athletes’ clinical symptoms of stress disorder. For example, the results showed that the most severe symptom of ACL rupture was avoidance-related [19], while the most troublesome problem for athletes who had concussions was sleep disorder [26]. The study showed that concussions could lead to neurobiological changes related to sleep–wake mechanisms. Sleep disorder has been proved to be one of the main symptoms described by injured patients after a concussion diagnosis [28]. This may also be considered an early sign of traumatic stress disorders.

Additionally, researchers need to be aware that injury events may not necessarily impact athletes’ psychological well-being. However, research shows that witnessing, or even imaging, an injury event could be very influential. The real impact is of the process and athletes’ perceptions of the event, which they experienced, witnessed or heard of, rather than the diagnosis from medical professionals.

Experimental research was conducted by recruiting a group of healthy athletes. They were asked to read about the scenario of an injury situation. Further, the participants were given a psychical diagnosis randomly at the end of the reading, followed by an assessment of stress disorder. Findings indicated that athletes who predicted injuries for themselves were very likely to experience PTSD in approximately the next 6 months after reading the scenario. However, there were no significant differences between the groups labelled with different diagnoses, such as concussion, mTBI and no diagnosis [29]. Thus, this line of research implies that the injury experience itself may be more influential than the level of physical dysfunction to athletes’ psychological and physical well-being. Specifically, some sports injuries may not affect the recovery of physical function, but the psychological trauma caused by them is not negligible.

In addition to the injured athletes, those who witnessed or heard of injuries from others may also show stress disorder symptoms. Therefore, research has focused on perceived injury infection in team sports. One study selected same-age teammates who suffered injuries as the target sample. The research showed that, when trauma events occur in a team (e.g. if teammates were injured), they may cause abnormal emotional and stress reactions in other uninjured athletes [22].

### Q2. What diagnoses are currently available for sports injury-related traumatic stress disorder?

After screening, three qualitative studies discussed stress disorder diagnosis in athletes (Table 2). Aron *et al*. suggested that it is difficult to diagnose stress disorder in this specific population due to the distinct characteristics of professional athletes [30]. They may be using compartmentalization and dissociation to mask trauma-related symptoms. Specifically, they are more willing to believe that their bodies do not belong to them and ignore the pain to complete the skill requirements [31]. Moreover, they generally perceived that seeking psychological support indicates disgrace and low self-esteem. Kaier *et al*. [32] found that athletes have a stronger sense of shame than ordinary people. They treat emotional problems as a sign of cowardice and conceal their negative symptoms [33]. Therefore, athletes can cover up their symptoms unconsciously after injury.

**Table 2:** Researches related to question 2

**Reference**	**Diagnosis**
Aron CM et al. 2019 [30]	(1) Diagnostic basis: DSM-5 (2) The characteristics of athletes, including compartmentalization and dissociation, make the diagnosis more difficult. (3) The specific diagnosis and treatment of stress disorders in athletes have not been established.
Miller-Aron C et al. 2021 [35]	(1) Diagnostic criteria (2) Basic diagnostic tools (3) Possible difficulties in diagnosis (4) The scale results need to be discussed more comprehensively (5) Consider ethnic and cultural differences
Lynch JH. 2021 [37]	(1) The degree of difficulty in diagnosing athletes’ stress disorder (2) Tools for stress disorder screening (3) The importance of clinician’s experience in the diagnosis of stress disorder

*DSM-V* diagnostic and statistical manual of mental disorders

The DSM-5 and International Classification of Diseases (ICD-11) are the most recognised assessments in the clinic. Within those scales, the main diagnostic tools for ASD include Brief Interview for Posttraumatic Disorder (BIPD), Acute Stress Disorder Interview (ASDI), Acute Stress Disorder Scale (ASDS) and Stanford Acute Stress Reaction Questionnaire (SASRQ) [34]. Additionally, the assessment tools for PTSD are divided into the self-reported questionnaire and structured diagnostic scale. According to the measured aspects, the self-reported questionnaire is constructed. Further, the subjects are required to answer the questions in order, based on their actual feelings, e.g. using the Revised Impact of Event Scale (IES-R). The structured diagnostic scale includes the Structured Clinical Interview Table (SCID) and Clinician-Administered PTSD Scale (CAPS) [10].

The three selected qualitative studies mentioned here also adopted other subjective scales, such as the review of traumatic stress disorders mentioned the Primary Care PTSD Screen for DSM-5 (PC-PTSD-5), the Trauma Screening Questionnaire (TSQ), the Startle, Physically Upset by Reminders, Anger and Numbness (SPAN) and the Short Posttraumatic Stress Rating Interview (SPRINT) [35].

The International Olympic Committee (IOC) developed an early identification tool for mental disorders for athletes, the Sport Mental Health Assessment Tool 1 (SMHAT-1), which includes items for PTSD. It can be used for early screening [36]. The previous study suggested that PTSD is a highly heterogeneous condition. It means that the diagnosis criteria of DSM-5 cannot accurately capture all trauma-related symptoms. Meanwhile, athletes live in conditions of high-intensity exercise and pressure in both training and competition, making it harder to recognise the specific symptoms of stress disorder related to sports injuries. Therefore, the self-reported scale cannot be used as the evidence or instrument in diagnosis, and clinical experience from practitioners is vital in ameliorating this problem [37].

### Q3. What kinds of rehabilitations are available for trauma-related symptoms in sports injury? How efficient are they in alleviating these symptoms?

Three articles on the rehabilitation of traumatic symptoms were included in the group (Table 3). Two of them are quantitative studies and the other was a case study.

**Table 3:** Psychological rehabilitation treatment

**Reference**	**Age, years**	**Sample**	**Measurements**	**Treatment**	**Result**
Mankad A et al. 2009 [39]	Average 21.93	15 Men 7 Women 8	IES POMS Becton Dickinson Immunological parameters reagent immune assay protocol	Writing sessions over three consecutive days	Pre-intervention (baseline): T1–T3 Three-day intervention: T4 Post-intervention (follow-up): T5–T6 (1) Participants reported high scores of intrusive thoughts and avoidance behaviours and showed a slow decline during the baseline time point. (2) After intervention T4, the score immediately showed a rise. However, 3-days intervention appeared to hasten the decline in individuals according to the slopes for intrusion and avoidance.
McArdle S. 2010 [7]	18	Men 1	/ (Case study)	(1) Dispute (2) Informal modelling (3) Systematic self-desensitization (4) Time projection	After the 4-month initial meeting, the athlete indicated that his flashbacks had decreased considerably.
Chen F et al. 2022 [40]	19–23	64	SASRQ	(1) Supporting (2) Cognitive-behavioural intervention (3) Relaxation training (4) Social support	The SASRD score was significantly lower than the scores post-intervention.

*IES* Impact of Event Scale, *A-POMS* Abbreviated Profile of Mood States, *CD-RISC* Connor-Davidson Resilience Scale, *SASRQ* Stanford Acute Stress Response Questionnaire

The common types of psychotherapies for stress-related disorders include cognitive behavioural therapy (CBT), cognitive processing therapy (CPT), prolonged exposure (PE) therapy and eye movement desensitization and reprocessing (EMDR) [38]. Treatments for injured athletes are also developed from these therapies. For example, one study attempted to verify the effect of prolonged exposure on athletes’ stress disorder symptoms. The experiment required the injured athletes to participate in a 3-day writing intervention. Each writing task consisted of two steps. Participants were first asked to recall their traumatic memories and pay attention to their feelings for 5 min. Then, they need to write their negative thoughts and feelings associated with the injury experience continuously for 20 min. The results showed that writing emotional disclosure can accelerate the decline of intrusion and avoidance [39]. Another empirical study implemented a comprehensive psychological intervention, including preoperative management, postoperative support, and other measures, which resulted in a significant decrease in the scores of athletes with acute stress disorders [40]. Additionally, another case study incorporated a variety of CBTs to help an 18-year-old male soccer player, including dysfunctional thoughts and images being disputed by systematically questioning the reality of the thought and image. The goal was to make them believe that full recovery is possible. First, the player was asked to think about or talk to other athletes and soccer players who had successfully returned to the sport after ACL reconstruction. Next, the athletes were asked to develop a fear hierarchy in order to cure the fear of re-injury and posttraumatic stress. Finally, the anxiety of re-injury and traumatic symptoms were addressed through self-desensitization. After 4 months of treatment, the athletes reported relief from posttraumatic stress [7].

Additionally, some researchers indicated that EMDR is the best treatment for PTSD related to athletic injury [5]. This guides the patients to constantly recall traumatic events, activate individual inherent information processing systems, accelerate nerve conduction activity, make the posttraumatic experience be repeatedly processed many times and adaptively reduce stress disorder symptoms [41]. In sports psychology, EDMR is considered an effective method for coping with negative emotions [42], relieving stress and restoring exercise levels. However, the therapeutic effect on stress disorder after sports injury still needs to be verified.

Specific treatment guidelines for stress disorder in the injured athletes’ population have not yet been formed. The current treatments are no different from those in the general population. Further, verification of the treatment effect of universal treatment for athletes is still minimal. Therefore, the effectiveness of interventions cannot be judged.

## Discussion

### New management model

**Figure 2.**
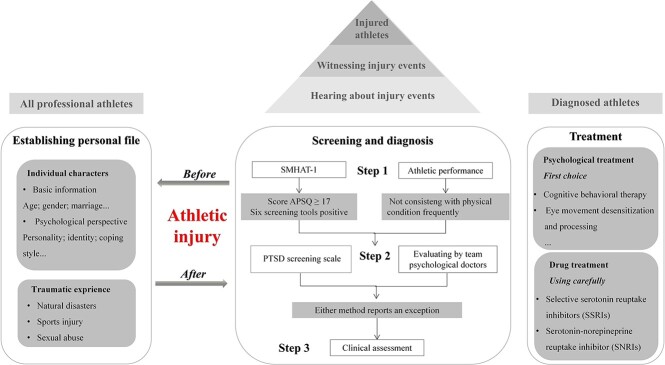
Traumatic stress disorder management framework after a sports injury. *SMHAT* Sport Mental Health Assessment Tool 1, *APSQ* Athlete Psychological Strain Questionnaire, *PTSD* post-traumatic stress disorder

Based on the existing findings, the present study attempted to develop a new framework (Figure **2**) for traumatic stress disorders after injury. Further, it explored a new in-depth direction in the field.

The literature related to Q1 proved that abnormal physical and psychological stress responses could occur after a sports injury. The severity and symptoms can be influenced by different factors, such as age, gender and psychological adjustment, found consistently across various studies. Conversely, a meta-analysis found that prior trauma experience is one of the critical predictors for PTSD, which should also be included in the assessment [43]. Therefore, identifying the potential risk of PTSD can be achieved by establishing a personal profile considering the factors mentioned above and providing guidelines for subsequent screening and diagnosis.

After a sports injury, primary screening and diagnosis are crucial. The timing of the treatment is key to the effectiveness of recovery. Furthermore, previous research highlighted that people diagnosed with ASD are more likely to develop PTSD [44]. Most patients with ASD have a good prognosis after prompt treatment, whereas patients with PTSD can suffer from these symptoms lifelong. Therefore, early screening should be carried out immediately after a sports injury to reduce the incidence of trauma-related stress disorder in athletes.

Both witnessing and experiencing traumatic events can lead to stress disorders; therefore, screening should not be limited to injured athletes. The negative emotion caused by sports injury may also affect other uninjured athletes. All the athletes aware of the incident should be considered [22].

The validity and reliability of the model developed by the Internal Olympic Committee, SMHAT-1, has been supported and used as an initial screening tool for mental disorders [36]. However, to reduce the influence of athletes’ hidden symptoms, subjective measurement is insufficient, and the clinical diagnoses of medical professionals and coaches are also necessary. For example, some athletes may avoid a particular motor skill in movement or exhibit a long-term mismatch between sports performance and physical state (dissociation). Therefore, both the self-report scale and observation should be considered in the diagnosis.

The currently available treatment tools are mainly drawn from drug therapy and psychotherapy. Considering the potential risk of drug therapy violating anti-doping policies, athletes should be cautious in their choice of drug treatment. For example, lamotrigine, an anticonvulsant drug aimed at intrusive symptoms and avoidance symptoms [45], is not prohibited by the World Anti-doping Code (WADC). However, the relevant regulations that allow international sports federations to ban specific drugs, may mean that anticonvulsant drugs are likely to violate the anti-doping regulations put forward by the World Archery Federation (WAF) [46].

Researchers suggested that psychological treatment should be the first choice for athletes with stress disorder and emphasised the effects of psychotherapy intervention on performance recovery [47]. A meta-analysis also confirmed this view, showing that psychotherapy was significantly superior to drug therapy in the follow-up study of PTSD. There were subtle nuances between psychopharmacological combined therapy and psychotherapy alone [48]. However, if the symptoms require pharmacological treatment, a combination of drugs can also be used. For instance, some medications such as selective serotonin reuptake inhibitors (SSRIs) and serotonin and norepinephrine reuptake inhibitors (SNRIs) can be prescribed to relieve symptoms related to stress disorders, and are allowed to be used by WADC and sports federations [37].

**Figure 3.**
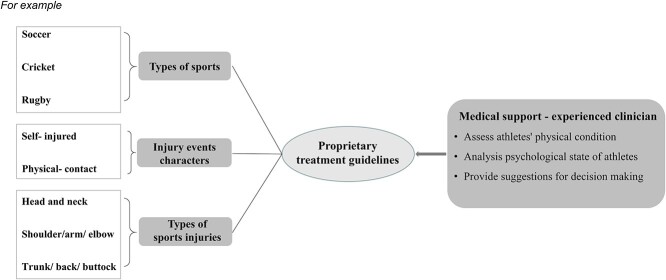
Proprietary treatment guidelines

Figure 3 highlights future research by developing a ‘tailored’ treatment guideline for sports injury-related PTSD. Athletes from different sports may have the same type of injury, but the impacts on their performance are different. For instance, ACL rupture (knee injury) may not be a career terminator for a rower, but it could be for a footballer. It implies that treatment must consider the sports type in which the injured athletes participated, the specific motor skills required by their sports and the characteristics of the injury event. A ‘tailored’ treatment means a guideline can be more specific to tackle issues unique to sports injury-related PTSD. For example, a volleyball player who suffered from an ankle sprain after blocking their symptoms of PTSD is different from a runner who twisted his ankle during routine jogging. The former injury is caused by inappropriate physical contact with others; the latter is caused by themselves. However, this line of research and practical guidelines have not been well developed, but the need for progress is urgent.

A more effective communication system between the coach and the medical team should be established to improve athletes’ stress disorder diagnosis and treatment [49]. Timely synchronization of information can ensure that both sides can better understand the physiological and psychological conditions of the athlete’s sports performance and improve the accuracy of judgment and decision-making. Additionally, it is necessary to provide an environment where athletes can express their psychological problems with coaching and medical team support.

### Review of the present research

The literature review of sports injury and stress disorder uncovered a considerable research gap in athletes’ stress disorder diagnosis and treatment systems. Therefore, researchers from diverse disciplines such as sports psychology, physiology and medical-related aspects, need to investigate this field further. Simultaneously, the depth and breadth of the existing research also need to be strengthened.

The research on stress disorder for injured athletes has the following problems. First, the pertinence is weak in that the majority of the literature does not analyse and evaluate stress disorder as an independent dimension but chooses to discuss it with other psychological problems. Thus, the accuracy of the evaluation cannot be guaranteed. Second, most research designs did not strictly control the impact of the related variables. For example, in addition to sex, age and other essential characteristics, the type of sport, injury category and other factors would also affect the relationship between sports injury and stress disorder. Finally, only very few studies paid attention to the physiological indicators of the abnormal stress response. Therefore, the results of the studies were generally lacking scientific support.

Stress disorder after a sports injury is a tremendous challenge to the continuation of an athletic career. However, research on the diagnosis and treatment strategies for athletes’ stress disorder is very scarce. The diagnosis of traumatic stress disorder is still based on a subjective scale without considering the psychological characteristics. Moreover, there is a lack of quantitative research to verify the effect of a treatment plan. Thus, it is hard to determine the most suitable therapy for athletes.

### Future research direction

The injury of competitive athletes occurs from time to time, especially in sports with high physical function. For example, research showed that the injury rate of 13–16 years old male sprinters and hurdlers is as high as 94.7% over 4 years [50]. During the recovery period after injury, athletes are prone to adverse emotional reactions. If they cannot cope with the negative emotions effectively, it may lead to the emergence of a stress disorder.

To better manage traumatic stress disorders in athletes and help them return to competition, it is essential to explore the stress disorder of competitive athletes more systematically and scientifically, mainly by including more physiological variables in the investigation. This study suggests that future research might investigate the following issues.

First, when the sports function is fully recovered, what changes have occurred in the sports performance compared with that before injury? What role does stress disorder play in changing the performance? There is a lack of comprehensive understanding of the stress disorder caused by sports injury in scientific research. Practitioners often ignore that the poor level of performance demonstrated by those injured athletes who return to the competition is the explicit manifestation of stress disorder after injury.

Second, what are the varied symptoms of stress disorder from different sports injuries? Would the type of sport affect the level of stress disorder facing the same injury events? Stress disorder may be affected by various factors, but in the process of literature retrieval it was found that there was no research that had addressed the issues mentioned above. It is a topic worth exploring, as the results of related research can provide a theoretical and empirical basis for establishing screening, diagnosis and grading criteria for stress disorder of athletes.

Third, what abnormal physiological indexes indicate stress disorder? How can the severity of stress disorder be evaluated through physiological indicators? In the long run, to solve the problem of deviation in the subjective evaluation of stress disorder, research in this field needs to understand the relationship between sports injury and stress disorder from the perspective of biological mechanism and to provide more objective and scientific support for the construction of the diagnosis and treatment system.

Often, athletes tend to cover up their abnormal stress reactions, which increases the difficulty of diagnosing and screening stress disorder. However, after in-depth consideration of various factors and characteristics of injured athletes, it is believed that the current diagnosis and treatment can be improved. Simultaneously, the traditional psychological intervention treatments often focus on the recovery of sports performance. However, there is a lack of illness perception toward PTSD, particularly as the evaluation of an athlete’s recovery relies heavily on their sports performance rather than the non-sports-related symptoms of PTSD, such as sleep disorder and fear of re-injury.

## Conclusions

The related study on sports injury and stress disorder is the direction to be further explored. It is necessary further to refine the performance of athletes with stress disorders and improve the diagnostic and therapeutic guidelines. The researchers should consider whether the ultimate reason for athletes to retire earlier is the damage of sports function from the injury or the lack of self-efficacy caused by stress disorder, thus holding them back from reaching the peak performance level again. Notably, this is a problem that should be addressed with more scientific support.

## Authors’ contributions

SXY conceived the idea for the research. SC and DLS completed literature search and analysis. SXY and SC drafted the initial manuscript and all authors contributed to revision prior to submission.

## Conflict of interest

The authors declare that they have no Conflicts of interest.

## Supplementary Material

Quality_assesment_tkac017

## Abbreviations

ACL: Anterior cruciate ligament; AD: Adjustment stress disorder; ASD: Acute stress disorder; CBT: Cognitive behavioural therapy; EMDR: Eye movement desensitization and reprocessing; IES: Impact of event; PTSD: Post-traumatic stress disorder; SCR: Skin conductivity response; SMHAT-1: Sport Mental Health Assessment Tool 1; WADC: World Anti-doping Code.
